# Systemic Lenvatinib Therapy Combined with Locoregional Chemoembolisation and Hepatic Arterial Infusion Chemotherapy for Advanced Hepatocellular Carcinoma with Main Portal Vein Invasion: A Multicentre Retrospective Case—Control Study

**DOI:** 10.3390/cancers18111776

**Published:** 2026-05-29

**Authors:** Dedi Wu, Ze Song, Jun Tang, Hongfei Miao, Min Tian, Wenzhe Fan, Jiahang Du, Zhiyong Lu, Hao Zhang, Yingqiang Zhang

**Affiliations:** 1 Department of Interventional Radiology, The Seventh Affiliated Hospital, Sun Yat-sen University, Shenzhen 518107, China; 2 Department of Oncology, The Seventh Affiliated Hospital, Sun Yat-sen University, Shenzhen 518107, China; 3 Department of Vascular and Interventional Oncology, Southern University of Science and Technology, Shenzhen 518055, China; 4 Division of Vascular and Interventional Radiology, Department of General Surgery, Nanfang Hospital, Southern Medical University, Guangzhou 510510, China; 5 Department of Radiology, Hunan Provincial People’s Hospital, The First Affiliated Hospital of Hunan Normal University, Changsha 410005, China; 6 Department of Interventional Oncology, The First Affiliated Hospital, Sun Yat-sen University, Guangzhou 510080, China

**Keywords:** hepatocellular carcinoma, portal vein tumour thrombosis, transarterial chemoembolisation, hepatic arterial infusion chemotherapy, lenvatinib, survival

## Abstract

Background/Objectives: Management of hepatocellular carcinoma (HCC) with main portal vein (MPV) invasion is challenging. We aimed to explore the efficacy and safety of systemic lenvatinib therapy combined with potent locoregional transarterial therapy (e.g., chemoembolisation plus infusion chemotherapy) for the treatment of HCC with MPV invasion. Methods: A direct comparison of different therapeutic regimens through a retrospective matched case—control study was conducted to evaluate the survival benefits of lenvatinib monotherapy versus lenvatinib combined with transarterial chemoembolisation (Len-TACE) versus Len-TACE plus hepatic arterial infusion chemotherapy (Len-TACE-HAIC) with oxaliplatin, fluorouracil, and leucovorin. Between January 2022 and December 2024, consecutive patients with HCC and MPV invasion who received lenvatinib, Len-TACE, or Len-TACE-HAIC from multiple centres in South China were enrolled for this analysis. Overall survival (OS), progression-free survival (PFS), and the objective response rate (ORR) were compared across the treatment groups. Adverse events (AEs) related to treatment were also recorded. Results: A total of 169 patients were included in the study: 48 patients received lenvatinib as systemic treatment, 56 received Len-TACE for locoregional and systemic therapy, and 65 received Len-TACE-HAIC for intensified locoregional and systemic treatment. Patients in the Len-TACE-HAIC group achieved a significantly greater ORR (53.8% vs. 28.6% vs. 6.3%, *p* < 0.001) than those in the Len-TACE group and the Len group did. Consistently, Len-TACE-HAIC resulted in markedly improved PFS (median, 7.0 vs. 5.0 vs. 2.0 months; *p* < 0.001) and OS (median, 15.0 vs. 10.0 vs. 7.0 months; *p* < 0.001). The incidence of grade 3-4 AEs was comparable across the three treatment groups. Conclusions: The results demonstrated that lenvatinib combined with potent locoregional therapy, i.e., the Len-TACE-HAIC regimen, provided superior survival benefits with an acceptable safety profile in patients with HCC and MPV invasion.

## Simple Summary

Approximately half of patients with hepatocellular carcinoma (HCC) are diagnosed with portal vein tumour thrombosis, a condition associated with a poor prognosis, particularly when the main portal trunk is invaded. This retrospective study aimed to compare the efficacy of lenvatinib monotherapy versus lenvatinib plus transarterial chemoembolisation (Len-TACE) versus Len-TACE plus hepatic arterial infusion chemotherapy (Len-TACE-HAIC) for HCC with main portal vein invasion. The Len-TACE-HAIC treatment resulted in a significantly higher objective response rate (53.8%), longer median progression-free survival (7.0 months), and a longer overall survival (15.0 months) than the other treatments, while maintaining a comparable safety profile. The combination of potent locoregional therapy, TACE plus HAIC, and lenvatinib offers superior survival benefits with acceptable safety for patients with HCC and main portal vein invasion.

## 1. Introduction

Hepatocellular carcinoma (HCC) is a common malignancy and a leading cause of cancer-related mortality worldwide. Approximately half of patients are diagnosed at an advanced stage and present with multiple tumour nodules or portal vein tumour thrombosis (PVTT) [1,2]. The prognosis of these patients is extremely poor because of impaired hepatic blood supply and accelerated intrahepatic tumour progression, particularly in patients with invasion of the main portal trunk (Vp4), for whom the median overall survival (OS) is less than 4.0 months [3]. Consequently, the management of HCC with main portal vein (MPV) invasion remains clinically challenging.

Over the past decade, tyrosine kinase inhibitors (TKIs), including sorafenib and lenvatinib, have been recommended as first-line treatments for advanced HCC with PVTT [4]. Although favourable outcomes were observed in the REFLECT trial, the efficacy of lenvatinib monotherapy remains limited, with a median overall survival (OS) of 13.6 months [5]. Recently, the LAUNCH trial highlighted the role of combination therapy with lenvatinib and transarterial chemoembolisation (TACE) in achieving improved response rates, progression-free survival (PFS), and OS compared with lenvatinib alone [6]. These results suggest that the addition of TACE may effectively enhance disease control and exert synergistic effects with lenvatinib, thereby improving survival time in patients with advanced HCC. However, for patients with PVTT, TACE should be administered with particular caution because of its limited response rate and potential risk of liver dysfunction.

Recently, accumulating evidence has demonstrated that hepatic arterial infusion chemotherapy (HAIC) with oxaliplatin, fluorouracil, and leucovorin has significant therapeutic effects in patients with unresectable HCC, especially those with PVTT [1,7]. Furthermore, compared with TACE alone, the combination of TACE and HAIC results in better tumour response and survival outcomes in advanced HCC patients with PVTT [8,9]. Therefore, we hypothesised that a potent transarterial therapy, TACE plus HAIC in one session, combined with lenvatinib-based systemic therapy may represent a promising strategy for advanced HCC with MPV invasion. To evaluate this approach, we conducted a multicentre retrospective case—control study among patients receiving lenvatinib as systemic therapy, lenvatinib plus TACE (Len-TACE) as combined locoregional and systemic therapy, or Len-TACE plus HAIC (Len-TACE-HAIC) as intensified locoregional and systemic therapy, with the aim of assessing the safety and efficacy of the Len-TACE-HAIC regimen in terms of tumour response and survival time.

## 2. Materials and Methods

### 2.1. Patient Selection

The protocol was approved by the ethics committees of the participating institutions, and written informed consent was obtained from all the patients enrolled. Between January 2022 and December 2024, consecutive patients with newly diagnosed HCC involving the MPV were identified through the electronic medical record system. The diagnosis of HCC was established in accordance with the Chinese guidelines for HCC [1]. HCC with MPV invasion was confirmed by the presence of a low-attenuation intraluminal mass that expands the MPV or causes filling defects within it, as demonstrated by contrast-enhanced computed tomography (CT) or magnetic resonance imaging (MRI). The inclusion criteria were as follows: (a) age between 18 and 75 years; (b) Eastern Cooperative Oncology Group (ECOG) score of 0–2; and (c) Child—Pugh class A or B. Patients were excluded if they: (a) had a malignancy other than HCC; (b) had received prior treatments, including liver surgery, local interventional therapy, or systemic chemotherapy; (c) had severe comorbidities involving the cardiac, pulmonary, renal, neurological, haematological, or coagulation systems; or (d) had incomplete data. The patients who received the treatment protocol were allocated to three groups: Len, Len-TACE, and Len-TACE-HAIC.

### 2.2. Treatment Protocols

#### 2.2.1. Lenvatinib Treatment

Lenvatinib (Eisai, Tokyo, Japan) was administered orally at a dosage of 12 mg/day (body weight ≥ 60 kg) or 8 mg/day (body weight < 60 kg) [5], and treatment continued until disease progression or unacceptable adverse events (AEs). In the Len group, it was initiated within 3 days of HCC diagnosis. Dose modifications were guided by grade 3 or 4 toxicities according to the Common Terminology Criteria for Adverse Events (CTCAE) version 5.0 [10].

#### 2.2.2. Transarterial Treatments

For patients who received Len-TACE, lenvatinib treatment was initiated within 7 days after the first TACE. TACE procedures were performed by two interventional radiologists with more than 10 years of experience in the field. Briefly, a 5-Fr catheter was inserted selectively into the celiac and superior mesenteric arteries, followed by angiography to identify tumour-feeding vessels and evaluate portal vein patency. All chemoembolisation procedures involved superselective catheterisation of the tumour-feeding arteries using a microcatheter. During embolisation, drug-eluting beads (DEBs; CalliSpheres, Hengrui Medical; DC Bead, Biocompatibles) or a lipiodol emulsion (2–20 mL) mixed with epirubicin was slowly infused into the hepatic tumour via the microcatheter. In cases of significant arterioportal or hepatic venous shunting, embolisation using polyvinyl alcohol was performed before the administration of DEBs or lipiodol emulsion. If residual tumour blush persisted after chemoembolisation, additional embolisation with polyvinyl alcohol was applied to reduce tumour blood flow. On the basis of the tumour burden, complications, and follow-up findings, the embolisation endpoint, defined as blood stasis in the tumour-feeding arteries, was achieved during the second or third TACE procedure [6].

For patients who received Len-TACE-HAIC, lenvatinib treatment was initiated within 7 days following the first TACE and HAIC procedure. For HAIC, the microcatheter was not removed after chemoembolisation but was retained in the main tumour-feeding hepatic artery. Patients were subsequently transferred to the ward for continuous infusion of the following drugs through the indwelling microcatheter: oxaliplatin at 85 mg/m^2^ over 2 h; leucovorin at 400 mg/m^2^ over 1 h; and fluorouracil at 400 mg/m^2^ as a bolus followed by 2400 mg/m^2^ over 46 h [9].

Locoregional transarterial therapy was followed by an on-demand strategy. When viable tumours or new intrahepatic lesions were detected, the transarterial treatments were repeated until an objective response could no longer be achieved after at least two procedures. Treatment was delayed or the dose was adjusted for patients who experienced clinical deterioration and hepatic decompensation.

#### 2.2.3. Nutritional Management

Nutritional management was provided to enrolled patients as it may be associated with the enhanced treatment response and improved survival outcomes [11,12]. Prior to initiating antitumor therapy, medical nutritionists conducted baseline nutritional assessments and formulated individualised nutritional support plans. Nutritional status was monitored continuously, and interventions were adjusted in accordance with nutritional response.

### 2.3. Follow-Up and Assessments

Patient follow-up was conducted at approximately 4-week intervals after the initial treatment until death or the end of the study (30 June 2025). Each follow-up assessment included imaging examinations and blood tests, including contrast-enhanced CT and/or MRI and assessments of the complete blood count, coagulation parameters, α-fetoprotein level, and liver and renal function. In this study, tumour response was assessed at the first radiological evaluation two months after initial treatment, according to the modified Response Evaluation Criteria in Solid Tumors (mRECIST) [13]. At each centre, an independent radiologist with more than 10 years of experience evaluated the radiological response, and a senior radiologist made the final determination in uncertain cases. Treatment-related AEs were recorded using CTCAE 5.0.

### 2.4. Outcomes

- The objective response rate (ORR) was defined as the proportion of patients who achieved a complete response (CR) or a partial response (PR), and the disease control rate (DCR) was defined as the proportion of patients who achieved a CR, a PR, or a stable disease (SD) in each group.
- OS and PFS were defined as the time from baseline radiological assessment to death or disease progression, respectively, for patients in each group.
- The incidence of common AEs was recorded in each group.

### 2.5. Statistical Analysis

All the statistical analyses were performed using SPSS Statistics, version 26.0 (SPSS, Chicago, IL, USA). Categorical variables were presented as frequencies (%) and were compared using the Pearson chi-square test or Fisher’s exact test, as appropriate. Continuous variables were reported as medians with interquartile ranges (IQRs) and were analysed nonparametrically. Survival curves, including OS and PFS curves, were generated using the Kaplan–Meier method, and group differences were assessed with the log-rank test. All tests were two-sided, and a *p*-value < 0.05 was considered to indicate statistical significance.

## 3. Results

### 3.1. Baseline Characteristics

**Figure 1:**
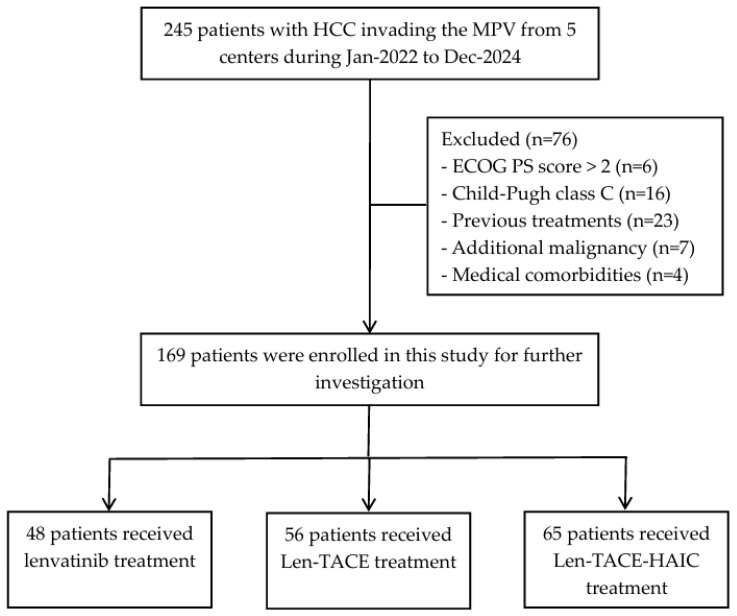
Flowchart of patient selection. HCC, hepatocellular carcinoma; MPV, main portal vein; ECOG PS, Eastern Cooperative Oncology Group performance status; Len, lenvatinib; Len-TACE, lenvatinib plus transarterial chemoembolisation; Len-TACE-HAIC, lenvatinib plus transarterial chemoembolisation and hepatic arterial infusion chemotherapy.

A total of 245 consecutive patients with HCC involving the MPV were retrospectively analysed between January 2022 and December 2024. According to protocol inclusion and violation, 169 patients were enrolled finally: 48 received lenvatinib monotherapy, 56 received Len-TACE, and 65 received Len-TACE-HAIC. The enrolment flow diagram was presented in Figure 1, and the baseline characteristics were summarised in Table 1. The study population was predominantly male, and majority of them had hepatitis B-virus-related HCC. Moreover, none of the variables differed significantly among the three groups with respect to ECOG performance, liver function, and tumour characteristics. Median duration of lenvatinib was 7.0 (IQR: 5.0–10.0) months in the Len-TACE-HAIC group, 5.0 (IQR: 3.3–7.0) months in the Len-TACE group, and 4.3 (IQR: 2.3–6.0) months in the Len group (*p* < 0.001). In addition, the median number of transarterial therapy cycles per patient was three (range, 2–6) in the Len-TACE-HAIC group, and four (range, 2–8) in the Len-TACE group.

### 3.2. Treatment Response

**Figure 2:**
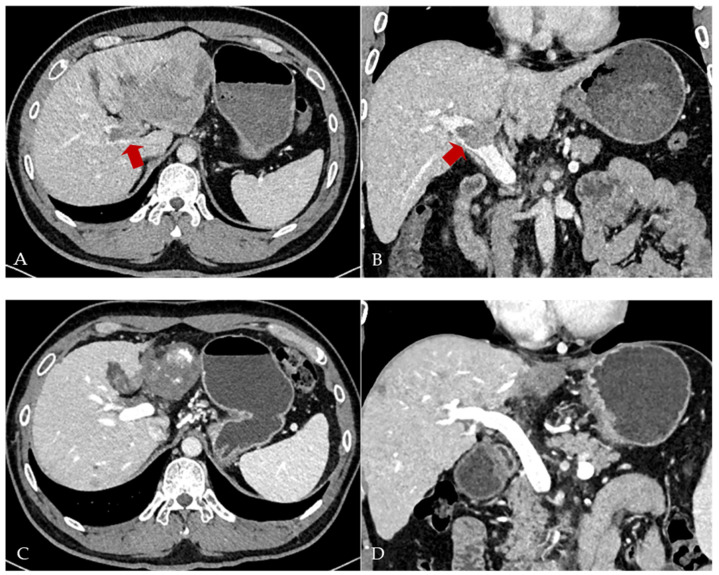
A 48-year-old man with hepatocellular carcinoma and major portal vein tumour thrombosis (PVTT) underwent combination therapy consisting of lenvatinib, transarterial chemoembolisation, and hepatic arterial infusion chemotherapy (Len-TACE-HAIC). (**A**,**B**) Pretreatment contrast-enhanced computed tomography (CT) revealed a 9.6 cm intrahepatic mass in the left lobe with major PVTT (arrow). (**C**,**D**) Follow-up contrast-enhanced CT performed 6 months after initial Len-TACE-HAIC demonstrated remarkable intrahepatic tumour shrinkage without enhancement and disappearance of major PVTT with patency of the main portal trunk.

After treatment, follow-up images at month 2 were available for all patients, and the results of tumour response evaluation across the three groups are presented in Table 2. Overall, significant differences were observed in the ORR (53.8% vs. 28.6% vs. 6.3%, *p* < 0.001) and DCR (81.5% vs. 66.1% vs. 47.9%, *p* = 0.001) among the Len-TACE-HAIC, Len-TACE, and Len groups (Figure 2). Intergroup comparisons revealed that the proportion of patients who achieved a CR or a PR was significantly greater in the Len-TACE-HAIC group than in the other groups. In addition, the proportion of patients who achieved a CR, a PR, or SD was also significantly greater in the Len-TACE-HAIC group than in the Len group, but no significant difference was observed compared with the Len-TACE group.

### 3.3. Overall Survival and Progression-Free Survival

**Figure 3:**
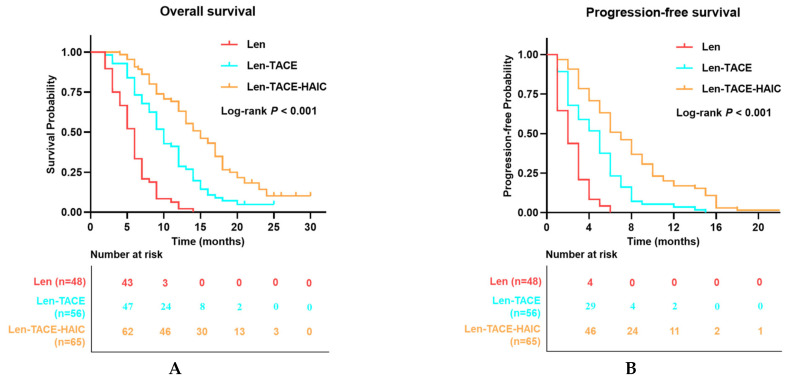
Kaplan—Meier curves for patients with advanced hepatocellular carcinoma and main portal vein invasion according to treatment modality. (**A**) The median overall survival was 15.0 months (95% CI: 12.0–18.0) in the Len-TACE-HAIC group, 10.0 months (95% CI: 8.7–11.3) in the Len-TACE group, and 7.0 months (95% CI: 5.2–6.8) in the Len group (*p* < 0.001). (**B**) The median progression-free survival was 7.0 months (95% CI: 5.6–8.4) in the Len-TACE-HAIC group, 5.0 months (95% CI: 3.8–6.2) in the Len-TACE group, and 2.0 months (95% CI: 1.3–2.7) in the Len group (*p* < 0.001). Len, lenvatinib; Len-TACE, lenvatinib plus transarterial chemoembolisation; Len-TACE-HAIC, lenvatinib plus transarterial chemoembolisation and hepatic arterial infusion chemotherapy.

At the end of follow-up (June 2025), 157 of 169 patients (92.9%) in the overall cohort had died, with a median follow-up of 14.5 months (range: 3–40 months). The median OS was 15.0 months (95% CI: 12.0–18.0) in the Len-TACE-HAIC group, 10.0 months (95% CI: 8.7–11.3) in the Len-TACE group, and 7.0 months (95% CI: 5.2–6.8) in the Len group. The median PFS was 7.0 months (95% CI: 5.6–8.4) in the Len-TACE-HAIC group, 5.0 months (95% CI: 3.8–6.2) in the Len-TACE group, and 2.0 months (95% CI: 1.3–2.7) in the Len group. Overall, patients who received Len-TACE-HAIC had significantly longer OS and PFS than those who received Len-TACE or lenvatinib monotherapy (*p* < 0.001; Figure 3). All pairwise comparisons of OS and PFS among the three groups revealed significant differences (all *p* < 0.001), confirming a significant survival benefit associated with Len-TACE-HAIC treatment.

### 3.4. Safety

All participants were included in the safety analysis, and the common AEs reported across the three groups are summarised in Table 3. The incidences of abdominal pain, fever, neutropenia, and thrombocytopenia were significantly higher in the Len-TACE-HAIC and Len-TACE groups than in the Len group. However, there was no significant difference in the incidences of all grade 3/4 AEs among the three groups. 

## 4. Discussion

In the present study, we compared the efficacy and safety of three treatment regimens, namely, lenvatinib vs. Len-TACE vs. Len-TACE-HAIC for HCC with MPV invasion. The results of the present study demonstrate that compared with the other treatments, lenvatinib combined with potent locoregional therapy (Len-TACE-HAIC) significantly improved tumour response and survival, with the highest ORR, PFS, and OS. Moreover, no treatment-related deaths occurred among the three groups, and all AEs were manageable with appropriate monitoring and interventions. These findings suggest that potent locoregional treatment could enhance the anti-tumour effect of lenvatinib and that Len-TACE-HAIC may be a better approach for treating HCC with MPV invasion.

The optimal treatment for advanced HCC patients with PVTT remains controversial. Although systemic lenvatinib therapy is recommended by several guidelines, such as those of the BCLC and NCCN, the efficacy of lenvatinib monotherapy is very limited, particularly in China, because of the high tumour burden observed in Chinese patients. Therefore, combination therapy incorporating intensive locoregional approaches such as TACE and HAIC is favoured as an initial treatment strategy [14,15]. For instance, adequate TACE combined with lenvatinib has demonstrated favourable efficacy and acceptable safety in patients with large intrahepatic tumours and PVTT [16]. However, this combination therapy is still approached cautiously in the context of MPV invasion because of concerns about liver function impairment and tumour recurrence. HAIC can deliver a sustained high concentration of chemotherapeutic agents directly into tumours, thereby enhancing the antitumour response with manageable AEs, and has been recommended for HCC patients with major PVTT [17]. Here, a novel perspective on the management of tumours with MPV invasion was provided, and our study confirmed a promising treatment pattern through a three-arm comparison: the Len-TACE-HAIC regimen achieved the highest ORR (53.8% vs. 28.6% vs. 6.3%, *p* < 0.001), PFS (median, 7.0 vs. 5.0 vs. 2.0 months, *p* < 0.001), and OS (median, 15.0 vs. 10.0 vs. 7.0 months, *p* < 0.001) compared with the Len-TACE regimen and lenvatinib monotherapy.

The benefits achieved by the Len-TACE-HAIC strategy may be attributed to the following reasons: (1) Powerful transarterial intervention promptly reduced the tumour burden. In the Len-TACE-HAIC group, residual tumours following incomplete embolisation in a single session, as well as PVTT supplied by the hepatic arteries, were exposed to high-concentration chemotherapeutic agents [7,18]. This approach may simultaneously reduce the risk of liver function impairment and chemotherapy resistance [19]. (2) Lenvatinib exerted a synergistic effect and enabled sustained tumour control. Lenvatinib significantly inhibited vascular endothelial growth factor and fibroblast growth factor, which are upregulated in the hypoxic environment by TACE [20], thus reducing tumour recurrence and metastasis [21]. Furthermore, improved vascular functionality, including reduced vascular leakage and enhanced permeability, as well as vessel normalisation induced by lenvatinib, may promote immune cell infiltration and the delivery of antitumour drugs [22,23]. (3) Because debulking of the tumour burden by TACE plus HIAC may improve liver function, the duration of lenvatinib treatment in the Len-TACE-HAIC group was significantly longer than that in the other groups (median, 7.0 vs. 5.0 vs. 4.3 months, *p* < 0.001), which enables a more durable and pronounced anti-tumour effect.

Previous studies reporting the efficacy of lenvatinib in combination with TACE and HAIC have reported an ORR of 61.2–68.5%, a median time to progression/PFS of 8.6–9.8 months, and an OS of 16.7–19.5 months [24,25], which were better than those observed in our cohort. A potential explanation for this discrepancy is that all the HCC patients included in our study were diagnosed with Vp4 PVTT, an indicator of a poor prognosis. Compared with segmental PVTT, MPV invasion increases the risk of intrahepatic metastasis and portal hypertension, thereby predisposing patients to gastrointestinal bleeding, ascites, and deterioration of hepatic function [26,27]. Thus, our study indicated that the Len-TACE-HAIC regimen can enable more patients, including those with Vp4 PVTT, to achieve superior outcomes and prolonged survival. Currently, TACE is recognised as a valuable treatment strategy for inoperable HCC and tumour down-staging, and it mainly includes conventional TACE (C-TACE) and DEB-TACE. While previous studies reported that DEB-TACE was associated with improved tumour response [28], particularly when smaller-diameter beads were used [29], the evidence was still insufficient to establish its superiority over C-TACE [15,30]. In this study, the selection of either C-TACE or DEB-TACE was determined by patient-specific factors, including tumour burden, liver function, and cost-effectiveness. Although it remains unclear whether the type of TACE influences the efficacy of combination therapy, our findings provide more individualised therapeutic options for HCC patients with MPV invasion.

Safety remains a primary concern in disease management. Abdominal pain, fever, neutropenia, and thrombocytopenia occurred more frequently in the groups receiving transarterial therapy, which was attributable to the use of chemotherapy drugs and embolic agents. However, the incidence of grade 3/4 AEs was comparable across the three groups. In addition, although no statistically significant differences were observed, the higher rate of AST/ALT elevation was notable in the Len-TACE-HAIC group than in the Len-TACE group and the Len group (50.8% vs. 35.7% vs. 31.3%, *p* = 0.079), which did not result in long-term liver dysfunction and may indicate improved treatment responses [31]. In summary, the combination of potent locoregional therapy with lenvatinib has favourable safety and tolerability profiles.

Several limitations of this study warrant consideration. First, comparative analyses among the three groups may be subject to selection bias because of the retrospective design. Second, although this was a multicentre study, the sample size remained limited, and the procedural quality of TACE and HAIC may vary across institutions, potentially affecting treatment consistency and outcome interpretation. Third, in the present study, the CT or MR imaging were used mixed, and although the evaluating efficacy of CT is slightly lower than that of MR, dynamic contrast-enhanced CT is still commonly used in imaging for evaluating the therapeutic efficacy of locoregional treatments, particularly for assessing iodised oil deposition and tumour viability after TACE. Therefore, large-scale prospective studies are needed to validate our results and enhance their generalisability.

## 5. Conclusions

Compared with lenvatinib monotherapy and Len-TACE therapy, potent locoregional therapy with TACE-HAIC combined with lenvatinib, i.e., Len-TACE-HAIC, resulted in improved treatment responses and survival outcomes, with a manageable safety profile for patients with HCC and MPV invasion.

## Figures and Tables

**Table 1:** Comparison of baseline patient characteristics.

Characteristic	Len (n = 48)	Len-TACE (n = 56)	Len-TACE-HAIC (n = 65)	*p*
Age, years, median (IQR)	55.0 (41.2–62.0)	52.5 (42.3–58.0)	50.0 (46.0–60.0)	0.374
Sex (n, %)				0.820
Male	44 (91.7)	53 (94.6)	61 (93.8)	
Female	4 (8.3)	3 (5.4)	4 (6.2)	
Aetiology (n, %)				0.906
Hepatitis B	41 (85.4)	48 (85.7)	55 (84.6)	
Hepatitis C	4 (8.3)	3 (5.4)	6 (9.2)	
Others	3 (6.3)	5 (8.9)	4 (6.2)	
ECOG PS score (n, %)				0.060
0	4 (8.3)	8 (14.3)	16 (24.6)	
1	44 (91.7)	48 (85.7)	49 (75.4)	
Child-Pugh class (n, %)				0.765
A	40 (83.3)	49 (87.5)	54 (83.1)	
B	8 (16.7)	7 (12.5)	11 (16.9)	
ALBI grade (n, %)				0.836
1	13 (27.1)	17 (30.4)	21 (32.3)	
2	35 (72.9)	39 (69.6)	44 (67.7)	
AFP (n, %)				0.136
≥400 ng/mL	36 (75.0)	35 (62.5)	37 (56.9)	
<400 ng/mL	12 (25.0)	21 (37.5)	28 (43.1)	
Main tumour size (n, %)				0.807
>10 cm	37 (77.1)	40 (71.4)	48 (73.8)	
≤10 cm	11 (22.9)	16 (28.6)	17 (26.2)	
Intrahepatic tumour number (n, %)				0.766
≥3	33 (68.8)	42 (75.0)	46 (70.8)	
<3	15 (31.3)	14 (25.0)	19 (29.2)	
Extrahepatic spread (n, %)				0.868
Yes	13 (27.1)	17 (30.4)	17 (26.2)	
No	35 (72.9)	39 (69.6)	48 (73.8)	

Data are numbers of patients; data in parentheses are percentages. Len, lenvatinib; Len-TACE, lenvatinib plus transarterial chemoembolisation; Len-TACE-HAIC, lenvatinib plus transarterial chemoembolisation and hepatic arterial infusion chemotherapy. IQR, interquartile range; ECOG PS, Eastern Cooperative Oncology Group performance status; ALBI, albumin-bilirubin; AFP, α-fetoprotein.

**Table 2:** Tumour response evaluated by mRECIST.

Tumour Response	Len (n = 48)	Len-TACE (n = 56)	Len-TACE-HAIC (n = 65)	*p*
Complete response (n, %)	0 (0.0)	0 (0.0)	0 (0.0)	
Partial response (n, %)	3 (6.3)	16 (28.6)	35 (53.8)	
Stable disease (n, %)	20 (41.7)	21 (37.5)	18 (33.8)	
Progressive disease (n, %)	25 (52.1)	19 (33.9)	12 (18.5)	
Overall response (n, %)	3 (6.3)	16 (28.6) ^1^	35 (53.8) ^1,2^	<0.001
Disease control (n, %)	23 (47.9)	37 (66.1)	53 (81.5) ^1^	0.001

Data are numbers of patients; data in parentheses are percentages; ^1^ indicates a significant difference in the data compared with the Len group; ^2^ indicates a significant difference in the data compared with the Len-TACE group. mRECIST, modified response evaluation criteria in solid tumours; Len, lenvatinib; Len-TACE, lenvatinib plus transarterial chemoembolisation; Len-TACE-HAIC, lenvatinib plus transarterial chemoembolisation and hepatic arterial infusion chemotherapy.

**Table 3:** Treatment-related adverse events.

Adverse Event, n (%)	Any Grade				Grade 3/4			
	Len (n = 48)	Len-TACE (n = 56)	Len-TACE-HAIC (n = 65)	*p*	Len (n = 48)	Len-TACE (n = 56)	Len-TACE-HAIC (n = 65)	*p*
Abdominal pain	21 (43.8)	39 (69.6) ^1^	48 (73.8) ^1^	0.002	5 (10.4)	8 (14.3)	10 (15.4)	0.736
Nausea/Vomiting	12 (25.0)	18 (32.1)	24 (36.9)	0.405	2 (4.2)	4 (7.1)	4 (6.1)	0.810
Fatigue	18 (37.5)	20 (35.7)	22 (33.8)	0.922	3 (6.3)	2 (3.6)	4 (6.1)	0.774
Diarrhoea	15 (31.3)	14 (25.0)	18 (27.7)	0.777	3 (6.3)	2 (3.6)	3 (4.6)	0.813
Fever	6 (12.5)	27 (48.2) ^1^	30 (46.2) ^1^	<0.001	1 (2.1)	6 (10.7)	5 (7.7)	0.226
Hypertension	16 (33.3)	18 (32.1)	23 (35.4)	0.930	6 (12.5)	8 (14.3)	9 (13.8)	0.963
Hand-foot syndrome	13 (27.1)	14 (25.0)	18 (27.7)	0.942	1 (2.1)	0	2 (3.1)	0.434
Neutropenia	2 (4.2)	12 (21.4) ^1^	23 (35.4) ^1^	<0.001	0	1 (1.8)	3 (4.6)	0.263
Thrombocytopenia	4 (8.3)	10 (17.9)	22 (33.8) ^1,2^	0.003	1 (2.1)	1 (1.8)	3 (4.6)	0.601
Elevated AST/ALT	15 (31.3)	20 (35.7)	33 (50.8) ^1^	0.079	3 (6.3)	5 (8.9)	6 (9.2)	0.832
Hyperbilirubinemia	8 (16.7)	14 (25.0)	15 (23.1)	0.567	2 (4.2)	4 (7.1)	5 (7.7)	0.734
Hypoalbuminaemia	10 (20.8)	16 (28.6)	15 (23.1)	0.630	2 (4.2)	5 (8.9)	5 (7.7)	0.624
Ascites effusion	5 (10.4)	8 (14.2)	10 (15.3)	0.736	0	2 (3.6)	2 (3.1)	0.437
Bleeding	0	1 (1.8)	0	0.362	0	1 (1.8)	0	0.362

Data are numbers of patients; data in parentheses are percentages; ^1^ indicates a significant difference in the data compared with the Len group; ^2^ indicates a significant difference in the data compared with the Len-TACE group. Len, lenvatinib; Len-TACE, lenvatinib plus transarterial chemoembolisation; Len-TACE-HAIC, lenvatinib plus transarterial chemoembolisation and hepatic arterial infusion chemotherapy. ALT, alanine transaminase; AST, aspartate aminotransferase.

## Data Availability

The data of the current study are available from the corresponding author upon reasonable request.

## Abbreviations

The following abbreviations are used in this manuscript:

HCC	hepatocellular carcinoma
MPV	main portal vein
PVTT	portal vein tumour thrombosis
TKIs	tyrosine kinase inhibitors
OS	overall survival
PFS	progression-free survival
TACE	transarterial chemoembolisation
HAIC	hepatic arterial infusion chemotherapy
ECOG	Eastern Cooperative Oncology Group
CTCAE	Common Terminology Criteria for Adverse Events
DEBs	drug-eluting beads
mRECIST	modified Response Evaluation Criteria in Solid Tumors
CR	complete response
PR	partial response
SD	stable disease
PD	progressive disease
ORR	objective response rate
DCR	disease control rate
IQR	interquartile range
